# Do cognitive measures and brain circuitry predict outcomes of exercise in Parkinson Disease: a randomized clinical trial

**DOI:** 10.1186/s12883-015-0474-2

**Published:** 2015-10-24

**Authors:** LA King, DS Peterson, M. Mancini, P. Carlson-Kuhta, BW Fling, K. Smulders, JG Nutt, M. Dale, J. Carter, KM Winters-Stone, FB Horak

**Affiliations:** Veterans Affairs Portland Health Care System, Portland, Oregon USA; OHSU Parkinson’s Center and Department of Neurology, School of Medicine, Oregon Health & Science University, 3181 S.W. Sam Jackson Park Rd., Portland, Oregon 97239-3098 USA; Knight Cancer Institute and School of Nursing, Oregon Health & Science University, Portland, Oregon USA

**Keywords:** Parkinson’s disease, Exercise, Brain imaging, and cognition

## Abstract

**Background:**

There is emerging research detailing the relationship between balance/gait/falls and cognition. Imaging studies also suggest a link between structural and functional changes in the frontal lobe (a region commonly associated with cognitive function) and mobility. People with Parkinson’s disease have important changes in cognitive function that may impact rehabilitation efficacy. Our underlying hypothesis is that cognitive function and frontal lobe connections with the basal ganglia and brainstem posture/locomotor centers are responsible for postural deficits in people with Parkinson’s disease and play a role in rehabilitation efficacy. The purpose of this study is to 1) determine if people with Parkinson’s disease can improve mobility and/or cognition after partaking in a cognitively challenging mobility exercise program and 2) determine if cognition and brain circuitry deficits predict responsiveness to exercise rehabilitation.

**Methods/Design:**

This study is a randomized cross-over controlled intervention to take place at a University Balance Disorders Laboratory. The study participants will be people with Parkinson’s disease who meet inclusion criteria for the study. The intervention will be 6 weeks of group exercise (case) and 6 weeks of group education (control). The exercise is a cognitively challenging program based on the Agility Boot Camp for people with PD. The education program is a 6-week program to teach people how to better live with a chronic disease. The primary outcome measure is the MiniBESTest and the secondary outcomes are measures of mobility, cognition and neural imaging.

**Discussion:**

The results from this study will further our understanding of the relationship between cognition and mobility with a focus on brain circuitry as it relates to rehabilitation potential.

**Trial registration:**

This trial is registered at clinical trials.gov (NCT02231073).

## Background

Falls due to balance and gait impairments are one of the most important health and quality of life issues in the elderly. Parkinson Disease (PD) is responsible for more falls than any other chronic disease and imposes a heavy burden on over 3 % of people over 65 years [1]. Until recently, gait and balance were largely perceived as automated, biomechanical processes that did not require cortical control. However, work over the past decade has demonstrated the important relationships between balance/gait/falls and cognition [2, 3]. In fact, 60 % of older people with cognitive impairment fall annually, approximately twofold more than cognitively intact peers, and the worse the cognitive deficits, particularly executive dysfunction, the more often people fall [4–6]. These studies support the notion that mobility and cognition are connected, perhaps because mobility relies upon common cortical-subcortical networks subserving cognition and balance. Executive function, defined as a set of higher order cognitive processes that control, integrate, organize and maintain other cognitive abilities, is often altered in people with PD. [7–9] Specific deficits include response inhibition, set switching and updating of working memory [10–12]. All of these executive components are required for functional mobility in everyday environments and the relationship between such function with balance and rehabilitation has not been explored in people with PD [2, 13].

Imaging studies also suggest a link between structural and functional changes in the frontal lobe (a region commonly associated with cognitive function) and mobility. A recent European study of 415 older people used diffusion tensor imaging (DTI) to show that parkinsonian signs of slow walking speed, and falls are related to white matter loss in the frontal cortex, but not the basal ganglia [14–16]. Likewise, a recent systematic review that included eighty-six published studies using various imaging modalities to relate neuroimaging to mobility reported that there were consistent finding across imaging modalities linking frontal lobe measures with mobility performance [17]. Specifically, this targeted review supports an increased cortical control of gait in aging, reduced volume in several regions of grey and white matter that relate to impaired mobility and consistent neuroimaging findings that reveal the basal ganglia, parietal and frontal cortices and cerebellum are related to mobility outcomes [17]. Recent work identifying the locomotor neural network, which includes the i) supplementary motor area, ii) subthalamic nucleus, iii) mesencephalic and iv) cerebellar locomotor regions, also provides evidence for reduced structural and altered functional connectivity in people with PD [18, 19].

Though mobility and cognitive function may be related, rehabilitation of such deficits typically remains separate. There is some evidence that cognitive training may improve motor function and that mobility training may improve cognitive function in the elderly but a recent meta-analysis on this topic revealed limited and low quality studies [20]. Although many individual studies of exercise and rehabilitation interventions report success in improving balance and gait in people with PD, the overall effect size of many interventions are sometimes minimal, often not reaching the minimally important change and/or minimal detectable change levels [20–25]. The limited success of rehabilitation treatment for mobility problems in people with PD may be because current physical therapy treatment does not directly address deficits related to frontal cortex dysfunction, such as cognition and cognitive control of balance and gait.

We recently documented that rehabilitation using the Agility Boot Camp (ABC) training resulted in multiple improvements in mobility such as turning, gait speed, sit-to-stand, and balance in people with PD. [26] With our increasing interest in the interaction of cognition and mobility, we adapted the ABC program to incorporate additional cognitive, particularly executive function, challenges known to be impaired in people with PD, now called Agility Boot Camp-Cognition (ABC-C). The purpose of this study is to 1) determine if people with PD can improve mobility and/or cognition after partaking in the ABC-C program compared to a control intervention of education and 2) determine if cognition and postural, cognitive, and brain posture/locomotor circuitry deficits predict responsiveness to the cognitively challenging Agility Boot Camp (ABC-C) rehabilitation. Our underlying hypothesis is that frontal lobe connections with the basal ganglia and brainstem posture/locomotor centers play a large role in postural deficits in people with PD and that postural impairments will be related to executive cognitive impairments. We will also determine which postural, cognitive and circuitry impairments predict efficacy of cognitively-challenging mobility rehabilitation.

## Methods/design

**Fig. 1:**
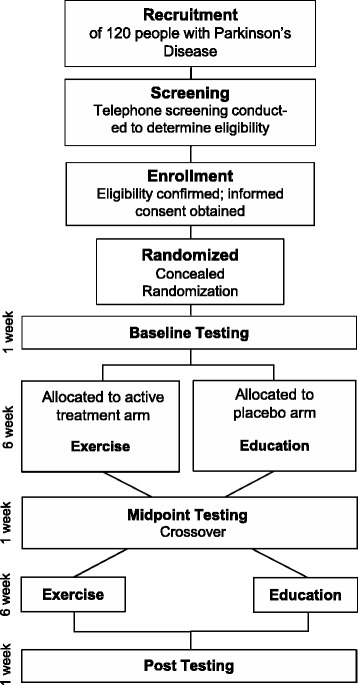
Consort diagram

The study is a cross-over, randomized, controlled trial design to determine responsiveness to the ABC-C exercise program. The study will include 120 people with PD (Fig. 1). People will be randomized into either an exercise (case) or education (control) 6-week intervention period. They will cross over after 6 weeks to receive the other treatment. Both interventions were designed to have the same frequency and will be delivered by the same exercise trainers for all sessions. Preceding the interventions, all patients will be tested on multiple measures of mobility cognition, and imaging. This same battery of tests will be administered after 6 weeks of intervention before the participants cross over into the second intervention. A final assessment will occur at the end of the second and final treatment arm (Table 2). All other interventions (medication, other interventions, exercise) will be kept as stable as possible and any changes in medication will be monitored. This trial is registered at clinical trials.gov (NCT02231073) and OHSU ethics committee has approved all aspects of the study. All research is in compliance with the Helsinki Declarations.

### Participants

Inclusion criteria for PD recruitment are a) ages 50–90 years old, without major musculoskeletal or peripheral or central nervous system disorders (other than PD) that could significantly affect their balance and gait, b) no recent changes in medication, excessive use of alcohol or recreational drugs, c) no history of structural brain disease, active epilepsy, stroke or acute illness, factors affecting gait such as severe joint disease, weakness, peripheral neuropathy with proprioceptive deficits, severe peripheral vascular occlusive disease, severe musculoskeletal disorders, uncorrected vision or vestibular problems, or dementia that precludes consent to participate or ability to follow testing procedures, d) ability to stand or walk for 2 min with or without an assistive device, e) no medical condition that precludes exercise, f) no claustrophobia, severe tremor, or any health history (implanted devices, Deep Brain Stimulation, etc.) that would interfere or put the subject at risk near the powerful magnetic field of the MRI scanner. Though the majority of participants will have the diagnosis of idiopathic PD a subset of people with frontal gait disorder may be included for pilot data analysis. Participants will also be assessed at baseline on the Montreal Cognitive Assessment as a global screening of cognition. All subjects will be tested at Oregon Health & Science University. All subjects will sign an informed consent and no minors will be included in the study.

### Idiopathic PD inclusion criteria

The United Kingdom Brain Bank criteria will be used. i.e., bradykinesia and at least one of the following: rest tremor (4–6 Hz), muscular rigidity, and postural instability not cause by visual, vestibular, cerebellar or proprioceptive dysfunction. Three or more of the following must be present for diagnosis of idiopathic PD: unilateral onset, rest tremor, progressive, persistent asymmetry, excellent response to levodopa, levodopa-induced dyskinesia [27]. All PD subjects will be Hoehn and Yahr Levels II-IV and responsive to levodopa.

### Sample Size

We computed power to detect differences between the intervention arms in a crossover trial via analysis of variance with level of significance set to 0.05 (SAS software v9.3). We assumed baseline levels and patterns of change similar to those reported in King 2013 [28]. We examined the significance of treatment effect from 1000 simulated replications of a crossover trial. The trial is comfortably powered (85 %) with 120 patients if total Mini-BESTest score increases by 2.4 points on average in the ABC arm, and only 1.3 points on average in the control arm. We predict a roughly 20 % drop-out rate, which is conservative based on our previous experience with short duration trials of rehabilitation in PD and by others [24, 25, 28, 29].

### Randomization and blinding

Subjects will be randomly assigned per centralized database; Research Electronic Data Capture (REDCAP) to either Education (ED) or Exercise (EX) first after passing the phone screening procedure. A computerized block randomization in will be centrally held in the redcap database-scheduling mode. Randomization will be implemented by an independent statistician using a block size of twelve subjects. The exercise trainer (unblinded) will notify the subjects by phone. The researchers who will be performing all pre, mid and post-tests will remain blinded to group assignment throughout the duration of the study.

### Intervention

#### Exercise

Subjects will participate in an 80-minute, group (6 per group) exercise session led by a certified exercise trainer knowledgeable in the ABC-C program for 3 days per week for 6 weeks. Trained research assistants will spot participates with high fall risk. Although the literature does not provide a clear dose–response for balance exercise intervention, there is consensus that a challenging program at higher doses (at least 2×/week) will show improvement [30, 31]. The exercise protocol is an adaptation of our ABC exercise program for PD (Table 1) [26] The theoretical basis for ABC is based on research from our laboratory and others that identified the primary neurophysiological and cognitive constraints that limit balance and mobility in PD [26, 28]. The exercises are designed as a circuit to challenge movement-skills known to be impaired in PD. Stations will include: (1) Gait training (2) PWR Moves © [32], (3) Agility course, (4) Lunges, (5) Boxing and (6) Tai Chi [33]. Each activity was chosen for its inherent focus on multi-directional movements, dynamic postural transitions, axial mobility, big movements and whole body motor sequencing. Each station is engaged for 10–20 minutes with rest periods in between stations. Each station is systematically progressed from beginning to intermediate to advanced levels by challenging: (1) divided attention with secondary cognitive tasks, (2) response inhibition, (3) limiting external sensory cues, (4) increasing speed and resistance.

**Table 1:** Overview of exercise and education interventions

EDUCATION		EXERCISE			
Frequency	One 90 min session/week, five 30 min relaxation sessions/week at home TOTAL: 240 min/week of education and relaxation	Frequency	Three 80 min sessions/week: TOTAL: 240 min/week of exercise		
Topic	Goal	Station	Time	Goal	Progression
Resources	Topic: Finding information on PD, communicating effectively with health care providers and building a support team.	Gait training	10	Warm up, big steps, arm swing	Speed, UE support (poles), cues for big steps, ankle weights, cognitive task
Sleep, Fatigue, Pain	Topic: Solutions to common sleep, pain and fatigue problem in PD.	PWR! Moves	20	Aerobic, whole body sequencing, amplitude training	3 levels of difficulty
Nutrition	Topic: Healthy eating guidelines, normal serving sizes, nutrition, and meal planning.	Lunging	15	Multi-directional, anticipatory control, dual task, executive function	Sequences, visual cues, UE support, cognitive task
Difficult emotions, stress and depression	Topic: Solutions to common emotional ups and downs that accompany chronic illness.	Agility	10	Turns, multi-directional, cognitive task	Speed, cognitive task
Communication	Topic: Improving communication (verbal, voice tone and body language).	Boxing	10	Powerful movements, dual task, anticipatory movement, executive function	Response inhibition, hand weights, cognitive task
Medication	Topic: Purposes, effects and responsibilities of common PD related medication.	Tai Chi	15	Weight shifting, limits of stability, postural responses, step initiation	3 levels of difficulty

#### Education

The Education program is a chronic disease education program to teach patients how to live better with their chronic condition. It was developed by our research team to be specific for people with PD. It will include content and discussion of topics such as sleep, nutrition, and medication management (Table 1). Classes will consist of a group of subjects (up to 6) meeting with the trainer for 90-minute session, once a week for six weeks. In order to match dose of the education intervention with the exercise intervention, participants will be provided relaxation tapes to be used at home 5 times per week for 30 minutes for an overall education dose of 240 minutes; similar to the exercise dose [34].

Compliance will be recorded at each session by the exercise trainer for both exercise and education. For the education arm, participants will record compliance for the relaxation sessions in a logbook. The trainer will code progression of exercise difficulty at the end of each week to determine the level of exercise progression for each person. Additionally, participants will state the level of perceived exertion (0–10 scale) after each exercise session. People will also wear hear rate monitor during class in order to assess the aerobic level of work being performed during exercise.

#### Assessment procedures

All people who are eligible per phone screening will come into the clinic for the informed consent process. An investigator will verbally explain the consent form, allow the person ample time to ready through the consent form and then will acknowledge consent by signing the form. All subjects will first read and sign consent forms. All outcomes will be measured in the practical OFF levodopa state (12 hours withdrawal). At baseline, people will be assessed on imaging, mobility and cognitive measures and will repeat the mobility and cognitive measures after the 1st six-week intervention and again after the 2nd six-week intervention (Table 2).

**Table 2:** Secondary outcome measures by domains

Domain Tested	Test	Description
Clinical		
	MDS-UPDRS	All sections of the Unified Parkinson’s Disease Rating Scale will be used to measure related to severity of PD.
	NFOGQ	The New FoG of Gait Questionnaire will be used to identify ‘freezers’ (score >3).
	Activities of balance confidence (ABC)	The ABC measures how balance confidence limits participating in the community.
	PDQ-39	This 39 item questionnaire measures multiple domains of quality of life.
Mobility		
	Balance	Postural sway during 30 seconds of quiet stance with and without cognitive task
	Turning	Smoothness of turning measured during the 1 min turning in place (360 degree turning in place) and turns during the 2 min walk with and without a cognitive task.
	Gait	Spatial and temporal gait metrics during walking with and without dual task
Imaging		
	High angular resolution diffusion imaging	High angular resolution diffusion imaging to assess white matter microstructure. Structural connectivity of the locomotor network will be assessed using probabilistic tractography.
	rsfcMRI	An indirect assessment of communication between spatially disparate neural regions. Analysis is restricted to neural regions comprising the locomotor network including the supplementary motor area, subthalamic nuclei, mesencephalic locomotor regions (pedunculopontine and cuneiform nuclei), and the midline cerebellar locomotor region
Cognition		
General	Scales for outcome of Parkinson’s Disease-Cognition (SCOPA-COG)	An instrument that was designed to assess the specific ‘frontal-subcortical” cognitive deficits found in Parkinson’s disease
Inhibition	Stroop task, flankers, Go/nogo, Stop signal task	The ability to deliberately inhibit dominant or prepotent responses when appropriate.
Shifting	Set-Shifting, Trail making task	The ability to flexibly alter behavior when relevant changes occur in the predefined goal or in the environment.
Updating	Dot counting task	The ability to update and monitor working memory representations.
Visuospatial	Benton judgment of line orientation test	The ability to identify a stimulus, its orientation, and its location.

### Primary outcome measure – Mini-BESTest

The primary outcome measure on which the intervention study was powered is the clinical *Mini-BESTest.* [35] The *Mini-BESTest* [35] is a sensitive measure of dynamic balance and includes 14 items (a maximum and best score of 28) [36, 37].

### Secondary outcome measures

Secondary measures for clinical measures, mobility (gait and balance), cognition and imaging domains are listed in Table 2.

### Clinical Measures

Our clinical tests will include assessment of quality of life, balance confidence, disease severity and freezing of gait. Specifically, the Parkinson Disease Questionnaire-39 (PDQ-39) [38] will be used for quality of life, the Activities-Specific Balance Confidence Scale (ABC) [39] will be used for balance confidence, the Movement Disorders Society Unified Parkinson’s Disease Rating Scale ( MDS UPDRS-Motor- Parts 1-IV) will be used to measure disease severity [13, 40] and the new freezing of gait questionnaire will be used as a self assessment of freezing of gait [41].

### Mobility Testing

All secondary measures of mobility come from performance of tasks while subjects are instrumented with body-worn, inertial sensors. Specifically, eight wireless, synchronized, Opal inertial sensors (APDM, Inc) will be applied with elastic Velcro bands to both feet, ankles and wrists, as well as the lumbar spine and mid sternum of the torso. Inertial sensor data collected at 128Hz will be wirelessly transferred to a laptop for automatic generation of gait and balance metrics by Mobility Lab and raw data for further analysis with Matlab [42]. Participants will perform tasks of quiet stance, the 2-minute walk test and the 360-degree turn test with and without a secondary, cognitive task (Table 2). All participants will wear a safety belt during the walking tasks and a trained research assistant will walk along side the participants. If a participant loses his or her balance, the research assistant will assist and prevent a fall. All safety measures will be taken to ensure a secure and comfortable environment. To prevent fatigue, participant will be repeatedly reminded that he or she may take a break whenever needed.

### Cognitive testing

Participants will complete a battery of cognitive tests to assess several dimensions of cognition. A table of all cognitive tests and a brief description is presented in Table 2. Most tests are categorized into the three domains of executive function defined by Miyake and colleagues (2000): *inhibition* (Stroop color-word test, Flankers, Go/NoGo, Stop Signal Reaction Time Test) [43–46], *set shifting* (Trail Making, Shifting Task) [45, 47–49] and *updating or working memory* (Dot Counting Test) [45]. We will also assess general cognition for people with PD via the Scales for Outcome of Parkinson’s Disease-Cognition (SCOPA-COG) and Montreal Cognitive Assessment (MoCA) [50, 51]. In addition, visuospatial function (Benton Judgement of Line Orientation) will be assessed.

### Imaging

The subject will be led into the MRI magnet room and positioned on the MRI system bed. A 32-channel Siemens rf-receiver coil will be placed appropriately for brain MRI, and the subject will be loaded into the magnet. During imaging, the subject’s head will rest on a special neck and head pillow to minimize head movements. Extra pillows under the knees and back will be used to make subjects as comfortable as possible. They will wear headphones to dampen noise during imaging and to allow subjects to hear and talk to the investigators at all times. Our procedures do not include administration of MRI contrast agents. The following protocol will then be executed:

Imaging data will be acquired using a 3.0 T Siemens Magnetom Tim Trio scanner with a 12-channel head coil. We will collect one whole brain high-resolution structural T1-weighted MPRAGE sequence (sagittal, TE = 3.58 ms, TR = 2300 ms, 256 x 256 matrix, resolution = 1 mm^3^, 1 average, total scan time = 9 min-14 s). We will also collect high angular resolution diffusion imaging using an echo-planar imaging sequence (72 different gradient directions, b-value = 3,000 mm/s^2^, TR = 7100 ms, TE = 112 ms, 2.5 mm^3^ voxels, 48 slices, FOV = 230 × 230 mm). Finally, we will acquire a resting-state functional MRI (*rs*-*f*MRI) using a gradient-echo echo-planar imaging sequence (TR = 2500 ms, TE = 30 ms, FA = 90°, 3.8 mm^3^volexs, 36 slices with interleaved acquisition, FOV = 240 × 240 mm). We will acquire two 10-minutes runs, providing a total of 20 minutes of resting state data for each subject in the study. Subjects will be instructed to lie still and keep their eyes open. Head padding will be provided to help subjects keep their heads still, earplugs will protect against scanner noise, and a leg bolster will be provided for back comfort. The technician will monitor the data and collect an extra scan if head movement > 1 mm is apparent.

#### Statistical Analysis

First, we will compare the amount of improvement in the Mini-BESTest with an analysis of covariance (ANCOVA) model, controlling for age. It is possible that lower functioning patients will have larger adaptation to ABC-C since they start out with less function. Alternatively, it is possible that the lower functioning patients will have poorer exercise tolerance so will improve less so we will also control for baseline Mini-BESTest*.* Because we are utilizing a crossover design, the treatment effect will represent change during the ABC-C versus control rehabilitation periods regardless of whether a patient received the ABC-C intervention during period 1 or period 2. We will assess, but do not anticipate, period by treatment group interaction effects. We will also enter an interaction term into the model to assess for differential intervention effects cognitive and/or frontal lobe structural and functional connectivity status. Second, we will relate the percent improvement in the Mini-BESTest with ABC-C intervention with baseline measures of posture/gait impairments, cognitive impairments and both structural and functional connectivity of the Posture/Locomotor circuit at baseline with linear regression models. Previous studies have shown the Minimal Detectable Change of the Mini-BESTest was 3 points [52]. We will also determine how many subjects move from high- to low-fall risk; a cutoff score for identifying PD fallers from non-fallers with the Mini-BESTest is 19/28 (63 %), with a sensitivity of .98 in 80 subjects [53].

## Discussion

The overarching goal of this study is to determine if cognitive function and frontal brain circuitry deficits predict responsiveness to exercise. Specifically, we are interested in understanding if certain phenotypes that best predict responsiveness to high intensity, short duration agility rehabilitation with a focus on cognition will help guide therapists to identify candidates for therapy and to develop specific therapy for specific types of mobility disabilities from parkinsonism. To date, there are very few studies on cognitive contributions to gait and balance as they relate to rehabilitation, particularly in this challenging population. Currently, physical therapists do not routinely incorporate cognitive challenges for people with PD and furthermore, it is unclear if these patients will benefit from such training.

If we find that executive function deficits and reduced structural and/or functional connectivity of the locomotor circuitry predict poor responses to challenging balance rehabilitation, that supports our hypothesis that frontal (and prefrontal) lobe impairments limits rehabilitation efficacy. If particular impairments of balance and gait improve more than others with ABC-C, this information will be used to improve the ABC-C intervention and will be followed by studies focused on determining which postural domains are most amenable to improvement with rehabilitation. The results from this study will further our understanding of the relationship between cognition and mobility with a focus on brain circuitry as it relates to rehabilitation potential.

## Abbreviations

ABC: Agility boot camp
ABC-C: Agility boot camp-cognition
ABC: Activities-specific balance confidence scale
ANCOVA: Analysis of covariance
DTI: Diffusion tensor imaging
ED: Education
EX: Exercise
MoCA: Montreal cognitive assessment
MDS UPDRS: Movement disorders society unified Parkinson’s disease rating scale
PD: Parkinson disease
PDQ-39: Parkinson disease questionnaire-39
REDCAP: Research electronic data capture
*rs*-*f*MRI: Resting-state functional MRI
SCOPA-COG: Scales for outcome of Parkinson’s disease-cognition
